# 作者更正

**Published:** 2024-02

《中华血液学杂志》2023年第44卷第11期第881页刊登的论文《血友病合并抑制物诊断与治疗中国指南（2023年版）》中的图3有误，“抑制物≤5 BU/ml”应为“抑制物<5 BU/ml”，“抑制物>5 BU/ml”应为“抑制物≥5 BU/ml”。特此更正。

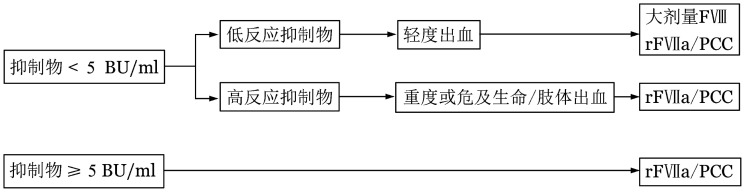



本文作者

